# Increased translocation of antigens to endosomes and TLR4 mediated endosomal recruitment of TAP contribute to nicotine augmented cross-presentation

**DOI:** 10.18632/oncotarget.9498

**Published:** 2016-05-20

**Authors:** Yan Yan Wang, Chun Fang Hu, Juan Li, Xiang You, Feng Guang Gao

**Affiliations:** ^1^ Department of Immunology, Basic Medicine Science, Medical College, Xiamen University, Xiamen, People's Republic of China; ^2^ State Key Laboratory of Oncogenes and Related Genes, Shang Hai Jiao Tong University, Shanghai, People's Republic of China

**Keywords:** mannose receptor, cross-presentation, α7 nicotinic acetylcholine receptor, dendritic cells, Toll-like receptor 4

## Abstract

Cross-presentation by dendritic cells (DCs) requires surface molecules such as lectin, CD40, langerin, heat shock protein, mannose receptor, mediated endocytosis, the endosomal translocation of internalized antigen, and the relocation of transporter associated with antigen processing (TAP). Although the activation of α7 nicotinic acetylcholine receptor (α7 nAchR) up-regulate surface molecule expression, augment endocytosis, and enhance cross-presentation, the molecular mechanism of α7 nAchR activation-increased cross-presentation is still poorly understood. In this study, we investigated the role of mannose receptor in nicotine-increased cross-presentation and the mechanism that endotoxins orchestrating the recruitment of TAP toward endosomes. We demonstrated that nicotine increase the expressiones of mannose receptor and Toll-like receptor 4 (TLR4) via PI3K-Akt-mTOR-p70S6 pathway. Both endosomal translocation of mannose receptor-internalized antigens and TLR4 sig- naling are necessary for nicotine-augmented cross-presentation and cross-priming. Importantly, the recruitment of TAP toward endosomes via TLR4-MyD88-IRAK4 signaling contributes to nicotine-increased cross-presentation and cross-activation of T cells. Thus, these data suggest that increased recruitment of TAP to Ag-containing vesicles contributes to the superior cross-presentation efficacy of α7 nAchR activated DCs.

## INTRODUCTION

In addition to classical MHC I -restricted endogenous antigen presentation [1], Surface molecules such as lectin, CD40, langerin, heat shock protein mediated cross-presentation allows dendritic cells (DCs) [2] present intracellular antigen and induce protective immunity against intracellular microbes infection or against tumors [3]. Nonneuronal cells such as DCs, epithelial cells and endothelial cells express nicotinic acetylcholine receptor (nAChR) [4]. Despite that nAChR activation promoted tumor metastasis and increased overall mortality [5–6], nicotine up-regulate surface molecule expressiones, augment DCs-dependent T cell activation on murine and human semi-mature DCs [7–11]. As model antigen ovalbumin (OVA) or tumor lysates do not originate from DCs, nicotine increasing DCs-dependent CTL priming indicates that α7 nAChR activation has positive effect on DCs cross-presentation, which occurs via vacuolar or endosome-to-cytosol pathway [12]. In the vacuolar pathway, antigens are degraded within endosomes by lysosomal proteases and loaded onto MHC I molecules [13]. In the endosome-to-cytosol pathway, receptors such as mannose receptor (MR) mediate antigen uptake, recruit internalized antigens toward endosomes [14–16]. Despite that the activation of α7 nAChR increases antigen internalization and promotes cross-presentation [7–11], the exact effect of α7 nAChR activation on MR expression and the mechanism of nicotine-increased cross-presentation are still uncertain.

In the endosome-to-cytosol pathway, the endosomal recruitment of the transporter associated with antigen processing (TAP) is essential for cross-presentation [14], as the internalized antigens in endosomes need to be transported from the endosomes into the cytosol [17] and the antigen-derived peptides in the cytosol still need TAP to retranslocate into endosomes [17]. Despite microbial molecular patterns, in particular Toll-like receptor (TLR) ligands [18], upregulate costimulatory molecule expression [19], TLR4-MyD88 signaling was demonstrated to mediate the endosomal relocation of TAP and Sec61 and permit the entry of antigenic peptides for cross-presentation [14, 20]. Despite that lipopolysaccharides (LPS) up-regulates co-stimulator molecules expression [21] and enables DCs to present antigens in the context of MHC I molecule [22–23], the exact effect and mechanism by which TLR4 signaling mediates the endosomal recruitment of TAP in α7 nAChR activation-increased cross-presentation is still to be clarified.

In the present study, we investigated the effect of nicotine on the expression of MR and TLR4, the role of MR in α7 nAChR activation-increased endosomal translocation of internalized antigens which subsequently activates T cells, and the mechanism of LPS orchestrating the endosomal recruitment of TAP. We demonstrated that nicotine up-regulate MR and TLR4 via PI3K-Akt-mTOR-p70S6 pathway. The increase of endosomal translocation of MR-internalized antigens, together with augmented recruitment of TAP toward endosomes via TLR4-MyD88-IRAK4 signaling, lead to α7 nAChR activation-augmented cross-presentation and thereby enhanced cross-activation of T cells.

## RESULTS

### Nicotine-increased mannose receptor expression via PI3K-Akt-mTOR-p70S6 pathway contributes to receptor-mediated endocytosis and the endosomal translocation of antigens

**Figure 1:**
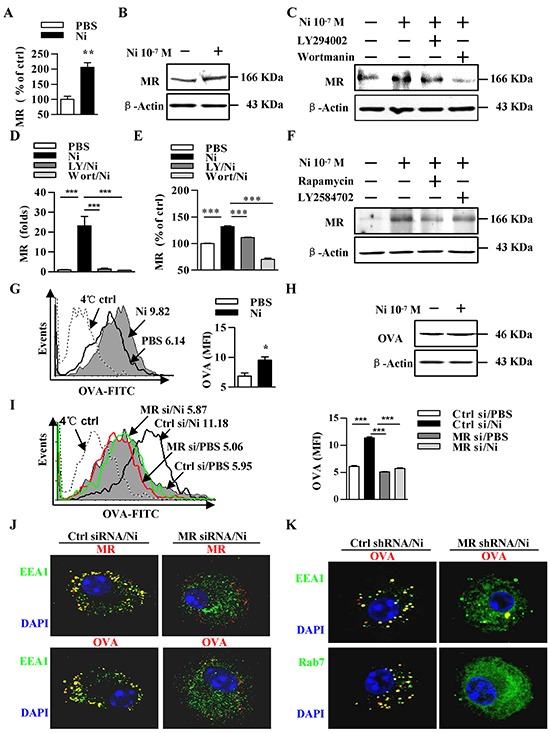
Nicotine-increased mannose receptor expression via PI3K-Akt-mTOR-p70S6 pathway contributes to receptor-mediated endocytosis and the endosomal translocation of antigens **A–F.** Murine DCs were pretreated with PBS or kinase inhibitors (10 μmol/l) LY294002, wortmannin, Rapamycin, LY2584702 2 h prior to nicotine (10^−7^ mol/l) 12~15 h stimulation. MR expression was determined via flow cytometry (A, E), western blot analyses (B, C, F) and Q-PCR (D). β-actin was used as an internal control. **G–K.** After pulsing with FITC-labeled (G, I) or unlabeled (H, J, K) ‘ordinary’ OVA (50 μg/ml), MR deficient and control DCs were conferred flow cytometric assay (G, I), western blot analyses (H), or Immunofluorescence (J-K) to monitor endocytosis (G-I) or antigenic endosomal translocation (J, K). For flow cytometry (G, I), numbers in histogram indicate MFI of analyzed population (left). Statistical analysis of MFI (right) is shown. The data are presented as the mean±SEM, *p<0.05, **p<0.01, ***p<0.001, student t test or one-way ANOVA with Newman-Keulspost test. One representative from 3 independent experiments is shown. Original magnification, ×600. Ni: nicotine; MR: mannose receptor; LY: LY294002; Wort: wortmannin; OVA: ovalbumin; si: siRNA.

Our previous studies showed that nicotine treatment promotes semi-mature DCs cross-priming [7–11]. The mechanism of antigen uptake determined the entrance of antigens into a specific intracellular pathway, which is required for efficient cross-presentation [24]. MR internalized OVA target into early-endosomes, inhibit endosomes’ mature into lysosomes [15]. The treatment with nicotine not only induced the activation of Erk and p38 (Supplementary Figure S1) but also promoted the phosphorylation of PI3K and increased co-stimulatory molecules expression [10–11]. Interestingly, the downstream kinases of PI3K, such as Akt, mTOR and p70S6, were efficiently activated with nicotine (Supplementary Figure S2). We incubated DCs with LY294002, wortmannin, rapamycin or LY2584702 prior to nicotine treatment and monitored MR expression. Whereas nicotine increased MR expression in both transcription level and translation level, the inhibitions of PI3K, Akt, mTOR, p70S6 kinases resulted in down-regulation of MR (Figure 1A–1F). The analyses of human PBMC-derived DCs also found nicotine increased MR expression via PI3K-Akt pathway (Supplementary Figure S3). As nicotine augmented α7 nAChR expression in murine [9] and human DCs (Supplementary Figure S4), the inhibition of α7 nAChR abolished nicotine's effect on MR expression (Supplementary Figure S4) indicates that α7 nAChR increase MR expression via α7 nAChR-PI3K-mTOR-p70S6 pathway.

Analyses of the intracellular antigen in nicotine-treated DCs revealed that nicotine increase about 60% abilities of antigen uptake (Figure 1G–1H). DCs could uptake soluble antigen OVA by MR-mediated endocytosis or pinocytosis, which is essential for cross-presentation or MHC II–restricted presentation, respectively [24]. We analyzed the *in vitro* uptake of OVA by MR-deficient DCs. While nicotine obviously increased DCs' ability of antigen uptake (Figure 1I), the MR deficiency (Supplementary Figure S5A-5D) abolished nicotine's effect on DCs uptake, indicating that MR mediate nicotine increasing DCs' endocytosis (Figure 1I).

We next analyzed whether α7 nAChR activation increases the endosomal translocation of antigens in the absence of the MR. Toward this end, we incubated MR deficient or control DCs with OVA. The data revealed that not only MR but also OVA co-localizes with EEA1, a marker of MR-targeted endosomes (Figure 1J). To demonstrate that nicotine-increased antigen internalization is indeed targeted toward endosomes, we analyzed the co-localization of OVA with EEA1 and Rab7. Importantly, the deficiency of MR not only decreased the co-localization of OVA with EEA1, but also reduces the internalization of OVA to Rab7 (Figure 1K), demonstrating that nicotine-enhanced uptake of OVA indeed is targeted into endosomes.

### Nicotine-increased TLR4 expression via PI3K-Akt-mTOR-p70S6 pathway augments cross-presentation

**Figure 2:**
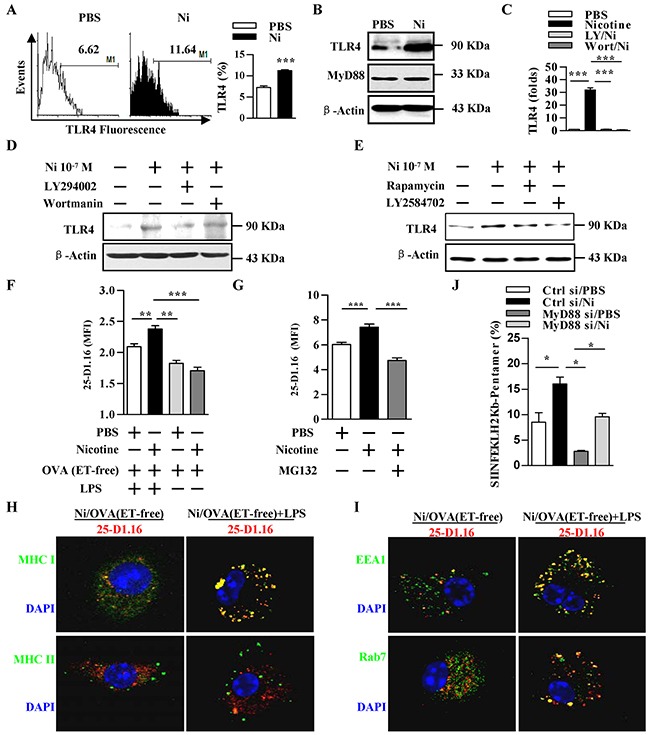
Nicotine-increased TLR4 expression via PI3K-Akt-mTOR-p70S6 pathway augments cross-presentation **A–E.** Murine DCs were pretreated with PBS or kinase inhibitors (10 μmol/l) LY294002, wortmannin, Rapamycin, LY2584702 2 h prior to nicotine (10^−7^ mol/l) 12~15 h stimulation. TLR4 expression was determined via flow cytometry (A), western blot analyses (B, D, E) and Q-PCR (C). β-actin was used as an internal control. **F.** Flow cytometric analyses of DCs previously exposed to ‘ordinary’ OVA or endotoxin-free OVA (OVA(ET-free)) with or without LPS exposure. **G.** Flow cytometric analyses of DCs conferred with PBS or proteasome inhibitor MG132 (20 μmol/l) 2 h prior to ‘ordinary’ OVA pulse. **H, I.** Immunofluorescence observation of nicotine-increased cross-presentation. Cross-presented OVA is stained with 25-D1.16 (red); MHC class I and II molecules are stained green (H); EEA1, Rab7 (all green); nuclei are counterstained with DAPI (blue). Original magnification, ×600. **J.** Flow cytometric analyses of OVA-specific CD8^+^ T cell priming in splenocytes of the recipients by SIINFEKL-H_2_Kb-pentamers staining. The data are presented as the mean±SEM, *p<0.05, **p<0.01, ***p<0.001, student t test or one-way ANOVA with Newman-Keulspost test. One representative from 3 independent experiments is shown. Ni: nicotine; TLR4: Toll like receptor; LY: LY294002; Wort: wortmannin.

Cross-presentation requires microbial endotoxins-induced DCs maturation [25] and TLR signaling-mediated MHC- I accumulation within phagosomes [26]. To elucidate the role of TLR4 in α7 nAChR-increased antigen cross-presentation, we treated DCs with nicotine and TLR4 expression was determined. The treatment with nicotine obviously increased TLR4's expression (Figure 2A–2B). The pretreatment with LY294002, wortmannin, rapamycin or LY2584702 abolished nicotine's effect on TLR4 expression in both transcription (Figure 2C) and translation level (Figure 2D–2E). The analyses of human DCs also found that nicotine increased TLR4 expression via PI3K-Akt pathway (Supplementary Figure S3). Interestingly, the inhibition of α7 nAChR with α-bungarotoxin or tubocurarine efficiently abrogated nicotine's effect on TLR4 expression (Supplementary Figure S6), indicating that nicotine increase TLR4 expression via α7 nAChR-PI3K-mTOR-p70S6 pathway.

Pathogen-derived antigens as well as model antigens ‘ordinary’ OVA often contain traces of endotoxins [5]. As endotoxin-free OVA is cross-presented far less efficiently than is ‘ordinary’ OVA [14], we wondered whether nicotine-increased cross-presentation would indeed need TLR4 signaling. To answer this question, we incubated DCs with endotoxin-free EndoGrade-OVA, either concurrently with short period (20 min) LPS (1 ng/ml) stimulation or for the same length of time but without LPS exposure. In LPS exposure condition, nicotine increased the efficiency of cross-presentation on endotoxin-free EndoGrade-OVA. But, without LPS exposure, the treatment with nicotine has no effect on cross-presentation and cross-priming at all (Figure 2F, 2J; Supplementary Figure S7). Importantly, nicotine-increased cross-presented OVA could also be inhibited by MG132 treatment (Figure 2G), demonstrating a requirement of proteasomes for nicotine-increased cross-presentation [24].

We next analyzed whether nicotine-increased cross-presented OVA is internalized into endosomes in the absence of TLR signaling. Toward this end, we incubated DCs with endotoxin-free EndoGrade-OVA, with or without short period (20 min) LPS (1 ng/ml) stimulation. Whereas short term LPS stimulation had no effect on the co-localization of cross-presented OVA with MHC II molecule, co-administration of endotoxin-free EndoGrade-OVA with short period LPS exposure resulted in enhanced co-localization of cross-presented OVA with MHC I molecule (Figure 2H). Importantly, in these cells, TLR4 signal increased the co-localization of cross-presented OVA with both EEA1 and Rab7 (Figure 2I), demonstrating that nicotine-enhanced cross-presentation indeed need TLR4 signaling.

### Nicotine-increased cross-presentation is dependent on the up-regulation of mannose receptor

**Figure 3:**
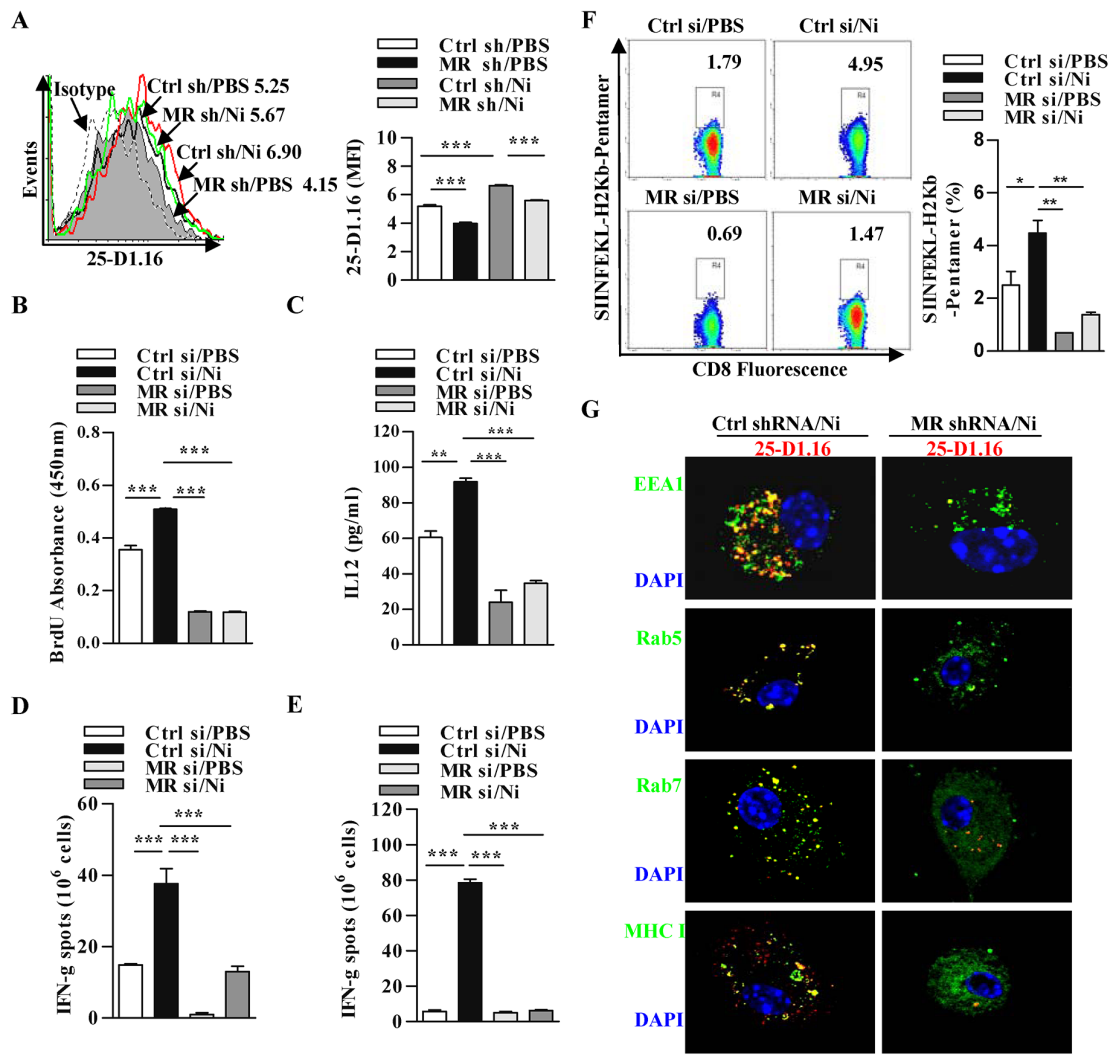
The up-regulation of mannose receptor is required for nicotine-increased cross-presentation MR deficient and control DCs were stimulated with nicotine and further incubated with endotoxin-free OVA with short term exposure of LPS. **A.** Flow cytometric determination of cross-presented OVA in DCs. Numbers in histogram indicates MFI of analyzed population. **B.** BrdU cell proliferation assay of splenocytes co-cultured with OVA-pulsed DCs. **C.** ELISA of IL-12 in supernatants of splenocytes co-cultured with OVA-pulsed DCs. IFN-γ Elispot assay of OVA-specific CD8^+^ T cells in the splenocytes **D.** and lymph nodes **E.** of the recipients which conferred intraperitoneal DCs transfer. **F.** Flow cytometric analyses of OVA-specific CD8^+^ T cell priming in splenocytes of the recipients by SIINFEKL-H_2_Kb-pentamers staining. Numbers in dot plot indicate positive percentages of analyzed population. **G.** Immunofluorescence observation of nicotine-increased cross-presentation. Cross-presented OVA is stained with 25-D1.16 (red); MHC class I, Rab5, EEA1, Rab7 (all green); nuclei are counterstained with DAPI (blue). Original magnification, ×600. The data are presented as the mean±SEM, **p<0.01, ***p<0.001, one-way ANOVA with Newman-Keulspost test. One representative from 3 independent experiments is shown. Ni: nicotine; MR: mannose receptor; si: siRNA; sh: shRNA.

Antigens internalization via pinocytosis or scavenger receptor-mediated endocytosis was rapidly targeted toward lysosomes for presentation on MHC II molecules [24]. If, antigens were internalized via MR-mediated endocytosis, they were routed into endosomes and processed for cross-presentation [14]. We demonstrated that nicotine increase endocytosis and augment the endosomal translocation of antigens; thus, we wondered whether MR up-regulation facilitates α7 nAChR activation increasing cross-presentation. To address this issue, we incubated nicotine-treated MR-deficient and control DCs with OVA, and accessed T cell proliferation and polarization. Given that siRNA transfection efficiently decreased MR expression (Supplementary Figure S5A-S5D), subsequent cytometric analyses demonstrated that nicotine-increased MR up-regulation indeed augmented cross-presented OVA in these DCs (Figure 3A). BrdU cell proliferation analysis and ELISA IL-12 determination further revealed that the up-regulation of MR not only increase DCs-dependent T cell proliferation but also promote Th1 polarization (Figure 3B–3C).

We next accessed the role of MR up-regulation in nicotine-increased cross-priming by the determination of antigen specific IFN-γ spot (Figure 3D–3E) or by flow analyses of SIINFEKL-H_2_Kb pentamers staining splenocytes (Figure 3F). Importantly, whereas nicotine increased the numbers of antigen specific IFN-γ spot, the MR down-regulation abolished the effect of nicotine on CTL priming in both splenocytes (Figure 3D) and lymph nodes (Figure 3E). The flow analyses of SIINFEKL-H_2_Kb pentamers positive splenocytes also revealed the similar conclusion (Figure 3F). All these observations demonstrate that nicotine-increased MR up-regulation is pivotal for α7 nAChR activation-augmented cross priming. In addition, the moderate cross-presentation was achieved in MR-deficient DCs, indicating that an alternative pathway of cross-presentation exist in these cells, which has already been observed by others [27].

Previous studies showed that early endosomes is the subcellular compartments for MR-internalized antigen targets into [28]. To investigate the role of endosomes in nicotine-increased cross-presentation, we incubated nicotine-treated MR-deficient and control DCs with OVA. Importantly, in these cells, MR down-regulation not only decreased the co-localization of cross-presented OVA with EEA1 and Rab5, but also reduces the co-localization of cross-presented OVA to Rab7 and MHC I molecule (Figure 3G), demonstrating that α7 nAChR activation-enhanced cross-presentation indeed takes place in endosomes.

### Nicotine-increased cross-presentation requires the endosomal recruitment of TAP via TLR4 signaling

**Figure 4:**
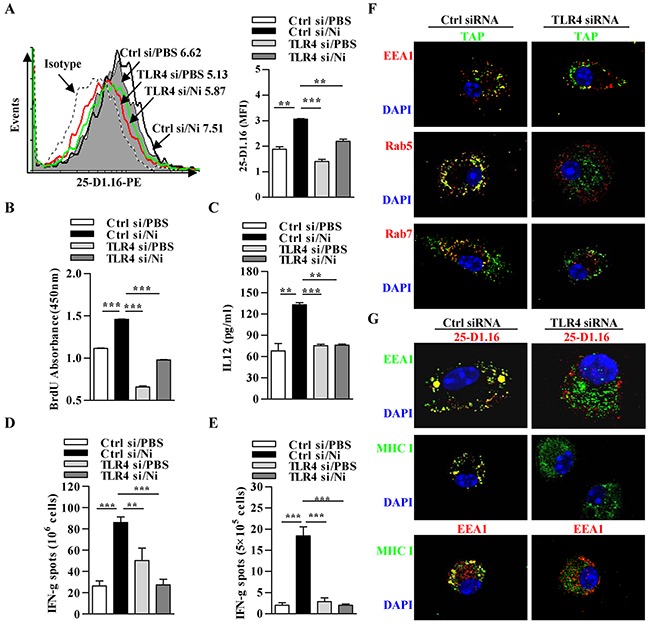
Nicotine-increased cross-presentation requires the endosomal recruitment of TAP via TLR4 signaling Nicotine-treated TLR4 deficient and control DCs were incubated with endotoxin-free OVA with short term exposure of LPS. **A.** Flow cytometric determination of cross-presented OVA in DCs. Numbers in histogram indicates MFI of analyzed population. **B.** BrdU cell proliferation assay of splenocytes co-cultured with OVA-pulsed DCs. **C.** ELISA of IL-12 in supernatants of splenocytes co-cultured with OVA-pulsed DCs. IFN-γ Elispot assay of OVA-specific CD8^+^ T cells in the splenocytes **D.** and lymph nodes **E.** of the recipients which conferred intraperitoneal DCs transfer. **F.** Immunofluorescence observation of the recruitment of TAP toward endosomes. TAP (green); Rab5, EEA1, Rab7 (all red); nuclei are counterstained with DAPI (blue). Original magnification, ×600. **G.** Immunofluorescence observation of TLR4 deficiency on nicotine-increased cross-presentation. Cross-presented OVA is stained with 25-D1.16 (red); MHC class I (green); EEA1 in 25-D1.16 co-localization is green and in MHC class I co-localization is red; nuclei are counterstained with DAPI (blue). Original magnification, ×600. The data are presented as the mean±SEM, **p<0.01, ***p<0.001, one-way ANOVA with Newman-Keulspost test. One representative from 3 independent experiments is shown. Ni: nicotine; TLR4: Toll like receptor; si: siRNA.

In phagosome-to-cytosol pathway, MR internalized antigens targeted toward endosomes and antigen-derived peptides were loaded onto MHC I molecules by endosomal TAP [29]. As nicotine increases TLR4 expression and augments proteasome-dependent cross-presentation (Figure 2), we wondered whether nicotine-increased cross-presentation needs the endosomal translocation of TAP. Toward this end, we incubated nicotine-treated TLR4-deficient and control DCs with OVA; and accessed cross-presented OVA, DCs-dependent T cell proliferation and polarization. Given that TLR4 expression was efficiently decreased (Supplementary Figure S5E-S5F, S5H), subsequent cytometric analyses revealed that nicotine-increased cross-presented OVA is indeed abrogated by TLR4 down-regulation (Figure 4A). BrdU cell proliferation analyses and ELISA IL-12 determination further revealed that the down-regulation of TLR4 signal not only decrease DCs-dependent T cell proliferation but also inhibit Th1 polarization (Figure 4B–4C). We next accessed the role of TLR4 signal in nicotine-augmented cross-priming by detecting antigen specific CTL priming (Figure 4D–4E). Importantly, nicotine obviously promoted antigen specific CTL priming in the recipients' splenocytes (Figure 4D) and lymph nodes (Figure 4E) in the existence with TLR4 signaling; whereas the inhibition of TLR4 signaling significantly abolished the effect of nicotine on antigen-specific cross-priming (Figure 4D–4E). All these observations demonstrate that nicotine-augmented cross-priming require TLR4 signaling.

In un-stimulated DCs, TAP is in the ER but not present in the endosomes [30]; whereas when DCs stimulated with LPS, a clear TAP translocation from the ER toward endosomes was observed [14], indicating that during DCs maturation, the transport of ER components to endosomes is tightly controlled [31]. To elucidate the role of TLR4 signaling in α7 nAChR activation-increased cross-presentation, we incubated nicotine-treated TLR4-deficient and control DCs with OVA, and accessed the endosomal recruitment of TAP and cross-presented OVA. Importantly, the down-regulation of TLR4 decreased nicotine-increased co-localization of TAP with EEA1, Rab5 and Rab7 (Figure 4F), demonstrating that nicotine-enhanced the recruitment of TAP toward endosomes indeed take place in TLR4 signal dependent manner. Likewise, the cross-presented OVA observation revealed that nicotine-enhanced cross-presentation is TLR4 signal dependent and indeed take place in endosomes (Figure 4G). All these observations demonstrate that α7 nAChR activation-augmented cross-presentation actually requires the endosomal recruitment of TAP via TLR4 signaling.

### Nicotine-increased cross-presentation requires the endosomal recruitment of TAP via MyD88 signaling

**Figure 5:**
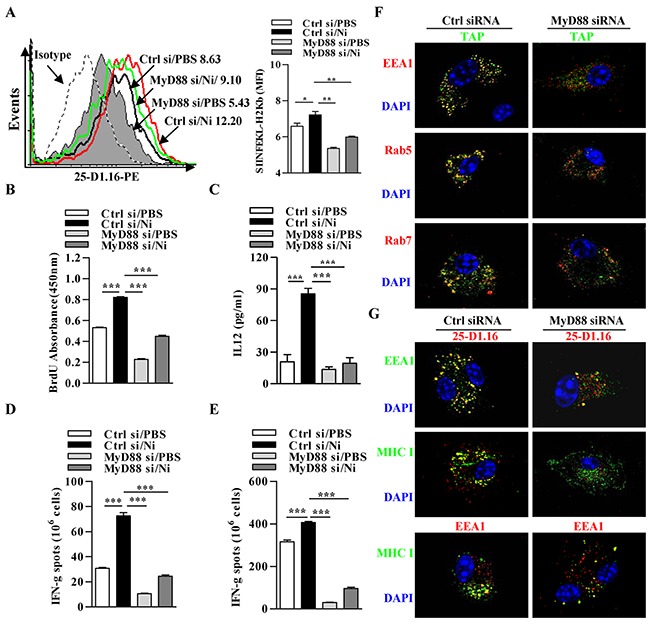
Nicotine-increased cross-presentation requires the endosomal recruitment of TAP via MyD88 signaling Nicotine-treated MyD88 deficient and control DCs were incubated with endotoxin-free OVA with short term exposure of LPS. **A.** Flow cytometric determination of cross-presented OVA in DCs. Numbers in histogram indicates MFI of analyzed population. **B.** BrdU cell proliferation assay of splenocytes co-cultured with OVA-pulsed DCs. **C.** ELISA of IL-12 in supernatants of splenocytes co-cultured with OVA-pulsed DCs. IFN-γ Elispot assay of OVA-specific CD8^+^ T cells in the splenocytes **D.** and lymph nodes **E.** of the recipients which conferred intraperitoneal DCs transfer. **F.** Immunofluorescence observation of the recruitment of TAP toward endosomes. TAP (green); Rab5, EEA1, Rab7 (all red); nuclei are counterstained with DAPI (blue). Original magnification, ×600. **G.** Immunofluorescence observation of MyD88 deficiency on nicotine-increased cross-presentation. Cross-presented OVA is stained with 25-D1.16 (red); MHC class I (green); EEA1 in 25-D1.16 co-localization is green and in MHC class I co-localization is red; nuclei are counterstained with DAPI (blue). Original magnification, ×600. The data are presented as the mean±SEM, *p<0.05, **p<0.01, ***p<0.001, one-way ANOVA with Newman-Keulspost test. One representative from 3 independent experiments is shown. Ni: nicotine; si: siRNA.

As the recruitment of TAP to endosomes depends on MyD88 but not on TRIF [18], the requirement of LPS stimulation (Figure 2F) and TLR4 signal (Figure 4) in nicotine-increased cross-presentation indicates that TLR4 signaling molecules MyD88 might be involved in α7 nAChR activation-increased cross-presentation. We incubated MyD88 deficient DCs (Supplementary Figure S5I-S5K) with OVA, and cross-presented OVA was determined by 25-D1.16 staining. Indeed, in the absence of MyD88, nicotine-increased cross-presented OVA was even lower than that obtained without nicotine treatment (Figure 5A). The findings that the absence of MyD88 alone achieves the lower cross-presented OVA than control DCs (Figure 5A) excluded the possibility that residual traces of endotoxin in the endotoxin-free OVA were responsible for its cross-presentation. Consistent with the results of cross-presentation, nicotine-increased DCs-dependent T cell proliferation and IL-12 secretion were completely abolished by MyD88 deficiency (Figure 5B–5C). The determination of antigen specific IFN-γ spot in splenocytes (Figure 5D) and lymph nodes (Figure 5E) also showed that the effect of nicotine on CTL priming was decreased by the deficiency of MyD88.

Finally, we studied whether MyD88 is needed to recruit TAP to endosomes and facilitate α7 nAChR activation-increased cross-presentation. TAP was localized together with endosome markers EEA1, Rab5 and Rab7 in nearly all wild-type DCs but in only very small population in DCs deficient in MyD88 (Figure 5F). These findings indicate that MyD88 signal is required for endotoxin-induced recruitment of TAP to endosomes. Likewise, 25-D1.16 staining revealed that nicotine-increased co-localizations of cross-presented OVA with EEA1 or MHC I molecule was inhibited by the deficiency of MyD88 signal, which indeed take place in early endosomal compartments (Figure 5G).

### Nicotine-increased cross-presentation requires the endosomal recruitment of TAP via IRAK4 signaling

**Figure 6:**
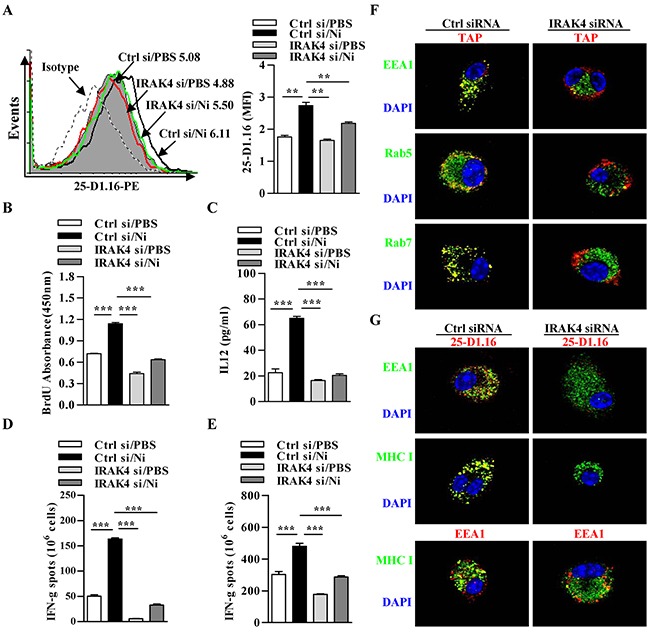
Nicotine-increased cross-presentation requires the endosomal recruitment of TAP via IRAK4 signaling Nicotine-treated IRAK4 deficient and control DCs were incubated with endotoxin-free OVA with short term exposure of LPS. **A.** Flow cytometric determination of cross-presented OVA in DCs. Numbers in histogram indicates MFI of analyzed population. **B.** BrdU cell proliferation assay of splenocytes co-cultured with OVA-pulsed DCs. **C.** ELISA of IL-12 in supernatants of splenocytes co-cultured with OVA-pulsed DCs. IFN-γ Elispot assay of OVA-specific CD8^+^ T cells in the splenocytes **D.** and lymph nodes **E.** of the recipients which conferred intraperitoneal DCs transfer. **F.** Immunofluorescence observation of the recruitment of TAP toward endodomes. TAP (green); Rab5, EEA1, Rab7 (all red); nuclei are counterstained with DAPI (blue). Original magnification, ×600. **G.** Immunofluorescence observation of IRAK4 deficiency on nicotine-increased cross-presentation. Cross-presented OVA is stained with 25-D1.16 (red); MHC class I (green); EEA1 in 25-D1.16 co-localization is green and in MHC class I co-localization is red; nuclei are counterstained with DAPI (blue). Original magnification, ×600. The data are presented as the mean±SEM, *p<0.05, **p<0.01, ***p<0.001, one-way ANOVA with Newman-Keulspost test. One representative from 3 independent experiments is shown. Ni: nicotine; si: siRNA.

Interleukin-1 receptor (IL1R)-associated kinase 4 (IRAK4), a downstream kinase of MyD88, was documented to induce the production of IFN-γ and IL-12 [32]. A reduced percentage of CD4^+^ and CD8^+^ T cell expressing IFN-γ was also observed in IRAK4 deficient mice [33]. These findings indicate that IRAK4 might be involved in TLR4 signaling inducing the endosomal recruitment of TAP. Toward this end, we incubated IRAK4-deficient and control DCs with OVA after nicotine stimulation and accessed cross-presented OVA by 25-D1.16 staining. Indeed, nicotine-increased cross-presented OVA was inhibited by the deficiency of IRAK4 (Figure 6A). Consistent with the decreased cross-presented OVA, nicotine-increased abilities of DCs-dependent T cell proliferation and IL-12 secretion were also diminished in IRAK4 deficient condition (Figure 6B–6C). Importantly, a reduced numbers of antigen-specific IFN-γ spot in splenocytes (Figure 6D) and lymph nodes (Figure 6E) were also achieved in IRAK4 deficient DCs transferred recipients.

We next accessed the effect of IRAK4 deficiency on endosomal recruitment of TAP and cross-presented OVA by immunofluorescence microscope. Importantly, in these cells, the deficiency of IRAK4 not only decreased nicotine-increased co-localization of TAP with EEA1, Rab5 and Rab7 (Figure 6F), demonstrating that endotoxin enhanced endosomal recruitment of TAP indeed takes place in IRAK4 dependent manner. Likewise, the observation of cross-presented OVA revealed that nicotine-enhanced cross-presentation is IRAK4 signal dependent and indeed take place in endosomal compartments (Figure 6G), indicating that IRAK4-mediated TLR4 signaling is crucial for α7 nAChR activation-increased cross-presentation and T cell activation.

### TLR4 signaling-promoted endosomal recruitment of TAP facilitates nicotine-increased cross-presentation

**Figure 7:**
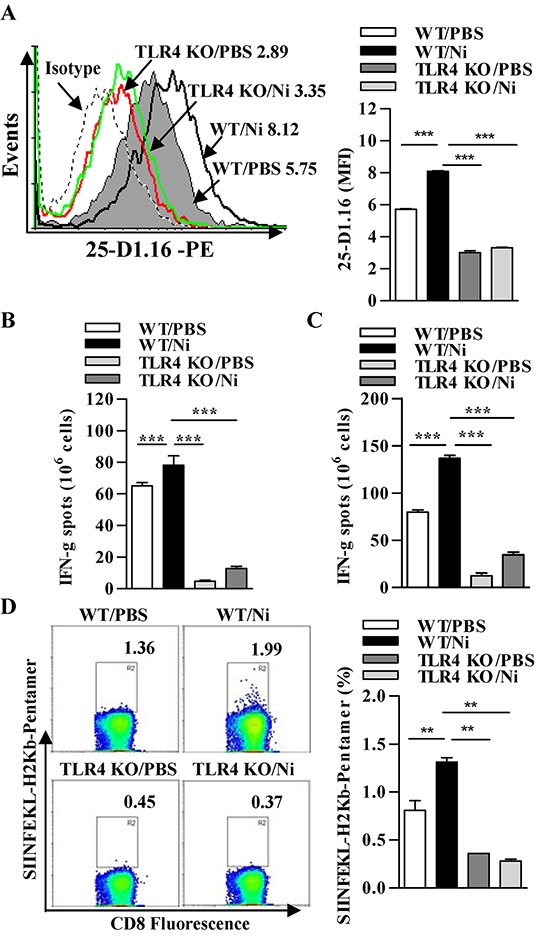
TLR4 signaling-promoted endosomal recruitment of TAP facilitates nicotine-increased cross-presentation Wild-type and TLR4 deficient DCs were stimulated with nicotine and further incubated with endotoxin-free OVA with short term exposure of LPS. **A.** Flow cytometric determination of cross-presented OVA in Wild-type and TLR4 deficient DCs. Numbers in histogram indicate MFI of analyzed population. **B-C.** IFN-γ Elispot assay of OVA-specific CD8^+^ T cells in the splenocytes (B) and lymph nodes (C) of the recipients which conferred intraperitoneal DCs transfer. **D.** Flow cytometric analyses of SIINFEKL-H_2_Kb pentramers positive cells in the splenocytes of DCs-transferred recipients. Numbers in dot plot indicate positive percentages of analyzed population. The data are presented as the mean±SEM, **p<0.01, ***p<0.001, one-way ANOVA with Newman-Keulspost test. One representative from 3 independent experiments is shown. Ni: nicotine; TLR4 KO: Toll like receptor 4 deficient; WT: wild type.

Since antigens targeted toward early endosomes by the MR are efficiently processed for cross-presentation [34], we examined whether nicotine-increased, MR-mediated endosomal translocation of OVA enhance cross-presentation in TLR4 deficient DCs. To address this object, the deficiency of TLR4 molecules in DCs of TLR4 knockout mice was firstly confirmed (Supplementary Figure S5G). Then, wild-type or TLR4 deficient DCs were incubated with OVA and cross-presented OVA was monitored. In TLR4 deficient DCs, the poor cross-presented OVA was observed; whereas in wild type DCs, strong cross-presented OVA could be achieved by the treatment with nicotine (Figure 7A). The determination of antigen specific CTL priming *in vivo* showed that nicotine increasing pronounced CD8^+^ T cell responses in splenocytes and lymph nodes were completely abolished in TLR4 KO mice (Figure 7B and 7C). Analyses of SIINFEKL-H_2_Kb pentamers staining splenocytes revealed that the effect of nicotine-increased CTL priming was abrogated in the condition that TLR4 signaling is absent (Figure 7D).

## DISCUSSION

**Figure 8:**
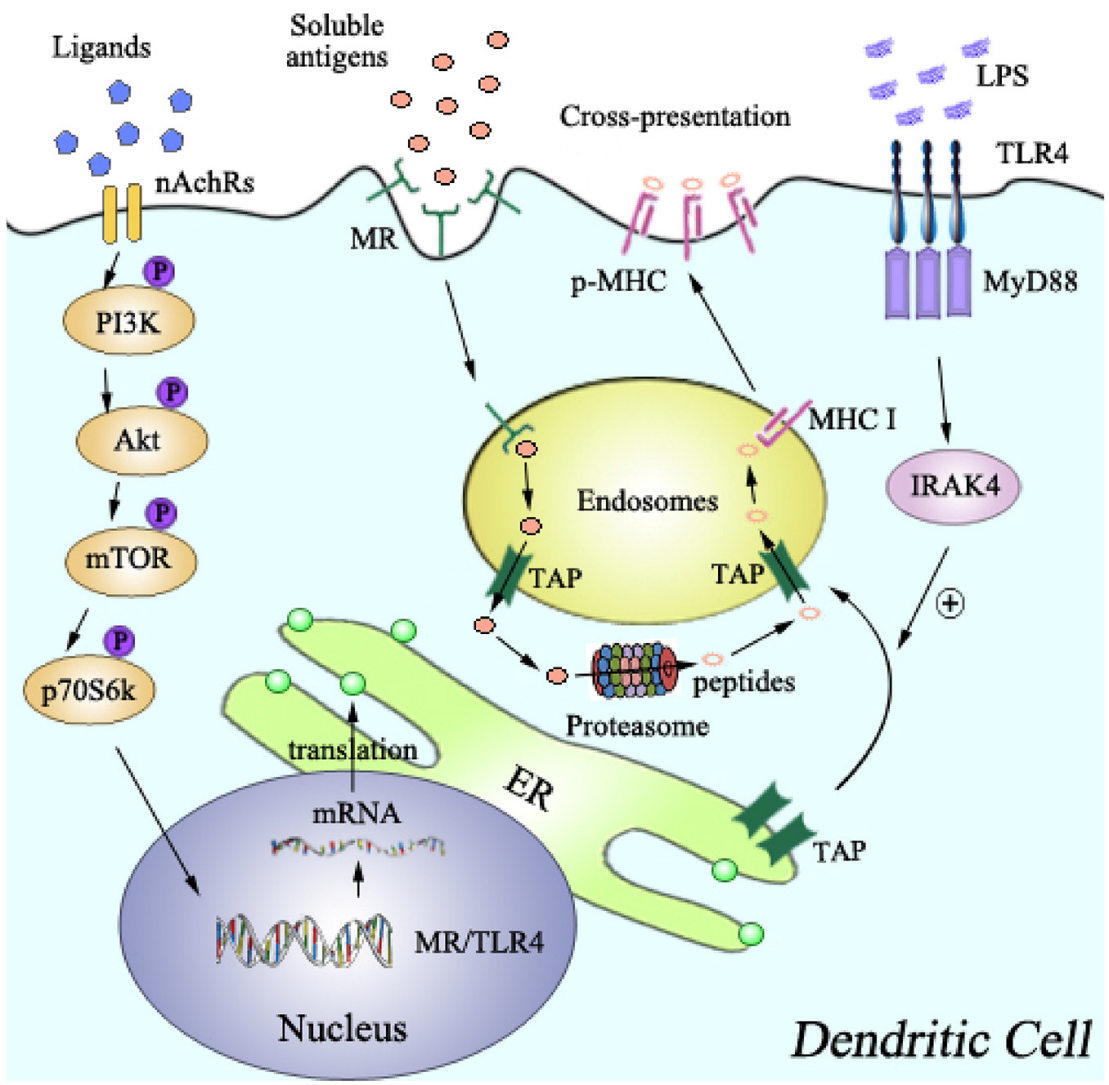
Model of mannose receptor and TLR4 signaling in nicotine-increased cross-presentation The up-regulation of MR and TLR4, which was achieved via PI3K-Akt-mTOR-p70S6 pathway, lead to the endosomal translocation of internalized antigens, and the endosomal recruitment of TAP via TLR4-MyD88-IRAK4 signal, respectively. Increased translocations of internalized antigens toward endosomes, together with the endosomal recruitment of TAP, facilitate α7 nAChR activation-increased cross-presentation and subsequent cross-priming.

In this study, we investigated the effects of increased MR and TLR4 signal in murine DCs on nicotine-enhanced cross-presentation and cross-priming. We demonstrated that nicotine increase the expressiones of MR and TLR4 via PI3K-Akt-mTOR-p70S6 pathway. Concurrently, MR up-regulation strongly enhances the endosomal translocation of internalized antigens; whereas TLR4-MyD88-IRAK4 signal efficiently promote the recruitment of TAP toward endosomes. Increased endosomal translocation of antigens combined with enhanced recruitment of TAP toward endosomes result in enhanced cross-presentation and thereby augmented the activation of antigen-specific T cells (Figure 8).

The finding that phagosomes containing the members of the MHC I loading machinery such as calreticulin, ERp57, tapasin, β2-microglobulin, Sec61, MHC I, and TAP, indicates that cross-presentation occurs in cellular compartments distinct from the ER [17]. Here, we have provided evidence that LPS-increased the recruitment of TAP toward endosomes is depended on TLR4-MyD88-IRAK4 signaling. Such the recruitment of TAP might represent a mechanism by which nicotine-increased cross-presentation can be restricted to microbial antigens carrying LPS. Meanwhile, other TLRs, such as TLR3, TLR7, and TLR9, which usually localize in the ER in unstimulated condition [35], are rapidly translocated toward endosomes upon TLR ligand stimulation [36]. Hence, the roles of other TLRs in nicotine-increased cross-presentation and whether TLR4 itself or the components of TLR4 signaling translocate to endosomes to induce such recruitment should be elucidated in further studies.

In our studies, nicotine-increased cross-priming is impaired by the deficiencies of TLR4 and its downstream molecules, indicating that TLR4 signaling could be regulated. IRAK-M was documented to inhibit the downstream signals of MyD88 [37], up-regulate IL-10 expression and lead to suppressed Th1 cell activation [38]. But, until now, the effect of α7 nAChR activation on the expression of IRAK-M remains uncertain and needs further exploration. While LPS increase CD4^+^ T cells activation by promoting phagosomes maturation [39] and augmenting lysosomal antigen processing [3]; the endosomal relocation of TAP via TLR4-MyD88-IRAK4 has been demonstrated to be essential for nicotine-increased cross-presentation (Figure 8), indicating that the components of cross-presentation ‘machinery’ can be recruited by TLR4 signaling [14]. As LPS increases the translocation of antigens to cytosolic proteasomes [40], therefore, in addition to increased recruitment of TAP toward endosomes, nicotine-increased TLR4 signaling might further act on proteasomes to regulate its abilities of antigen processing, which remains to be elucidated.

MR consists of an N-terminal cystein-rich domain, a fibronectin type II repeat domain, eight carbohydrate recognition domains (CRD), a transmembrane domain and a short intracellular region [41]. Through CRD4, the MR binds glycosylated proteins terminated in mannose, fucose or GlcNAc, and leads to their internalization [41]. In the present study, the up-regulation of MR induced by α7 nAChR activation not only increased antigen uptake but also promoted endosomal translocation of internalized antigens (Figure 1). Hence, there is no surprise to find that nicotine-promoted cross-presentation and cross-priming was efficiently abolished by the down-regulation of MR (Figure 3), indicating that the connection between MR and cross-presentation made the MR a promising subject for antigen targeting studies in approaches aimed at the induction of a strong cytotoxic T cell response [42].

Taken together, our data provide a new molecular mechanism for α7 nAChR activation-increased cross-presentation, which is mediated by the combined action of increased expression of mannose receptor and enhanced endosomal recruitment of TAP via TLR4-MyD88-IRAK4 signaling (Figure 8). This mechanism provides new insights into the molecular mechanisms of cross-presentation and might thus open new opportunities for therapeutic intervention in DCs-dependent T cell vaccine.

## MATERIALS AND METHODS

### Mice

Pathogen-free C57BL/6 mice (female, 6~8 weeks old) were bought from the Shanghai Laboratory Animal Center of Chinese Academy of Sciences (China) and kept at the Animal Center of Xiamen University. TLR4^−/−^ mice were provided by G. Jin (Xiamen University). This study was carried out in strict accordance with the recommendations in the Guide for the Care and Use of Laboratory Animals of the ARRIVE guidelines. The protocol was approved by the Committee on the Ethics of Animal Experiments of the Xiamen University.

### Reagents and antibodies

Reagents were purchased from the following companies: Nicotine (N3876), LPS from Escherichia coli was obtained from Sigma-Aldrich (St. Louis, MO, USA). DAPI was obtained from Vector Laboratories, Inc (Burlingame, CA, USA). Recombinant mouse GM-CSF and IL-4 were obtained from PeproTech (Rocky Hill, NJ, USA). Albumin from chicken egg white (OVA, Endotoxin-free EndoGrade-ovalbumin) was purchased from Hyglos GmbH (Regensburg, Germany). OVA peptide SIINFEKL of amino acids 257~264 were synthesized by Auspep (Tullamarine, VIC, Australia). LY294002 and wortmannin were from Cayman Chemical (Ann Arbor, MI, USA). Rapamycin, LY2584702, and MG132 were bought from Selleck Chemicals (Houston, TX, USA). Purified anti-ovalbumin antibody (TOSG1C6), PE-conjugated antibody anti-mouse H_2_Kb bound to SIINFEKL (25-D1.16), FITC-conjugated antibody to mouse MR (C068C2), mouse TLR4 (SA15-21), H_2_Kb (AF6-88.5), I-A/I-E (M5/114.15.2), FITC-conjugated OVA, as well as brefeldin A solution (BFA, 1000×), were obtained from BioLegend (San Diego, CA, USA). PE-conjugated SIINFEKL-H_2_Kb pentamers was bought from Proimmune (Oxford, UK). Antibody to TLR4 (D8L5W), MyD88 (D80F5), β-actin (13E5), were bought from Cell Signaling Technology (Beverly, MA, USA). BrdU cell proliferation kit was obtained from Roche (Roche Diagnostics GmbH, Germany); Fluorescence conjugated antibodies to murine CD8, MHC I and II molecules, IL-12 p70 ELISA kit (BMS6004) were obtained from eBioscience (San Diego, CA, USA). Antibody to MR (15-2, #ab8918), Rab5 (Rab5-65, #ab50523), Rab7 (Rab7-117, #ab50533), anti-Rabbit IgG (Chromeo 546, #ab60317), anti-Rabbit IgG (Chromeo 488, #ab60314), anti-Mouse IgG (Cy3, #ab97035), anti-Mouse IgG (Alexa Fluor, #ab150117), and anti-goat IgG (DyLight 488, #ab98514) antibodies were from Abcam (Cambridge, UK). TRI-zol was purchased from Invitrogen life technologies (Carlsbad, CA, USA). IFN-γ Elispot kit were obtained from U-CyTech Biosciences (Utecht, Netherlands). The siRNA of MR (sc-45361), TLR4 (sc-40261), MyD88 (sc-35987), IRAK4 (sc-45401) and control siRNA (sc-37007), antibody to MR (MR5D3), TAP (R-20), EEA1 (E-8), ovalbumin (2D11), anti-rat IgG (CFL 488, #sc-362263), anti-rat IgG (CFL 405, #sc-362253), was obtained from Santa Cruz Biotechnology (Dallas, TX, USA). The PrimeScript RT-PCR kit and SYBR Premix ExTaqTM kit were purchased from Tarkara Bio (Dalian, Liaoning, China). RPMI-1640 medium and fetal bovine serum (FBS) was purchased from HyClone (Logan, UT, USA).

### Generation of murine bone marrow-derived semi-matured DCs

Bone marrow-derived DCs were generated by culturing progenitors for four days [11] in RPMI 1640 medium supplemented with 10 ng/ml GM-CSF, 1 ng/ml IL-4, and 10% FBS. Non-adherent cells were gently washed out with PBS on day 4 of culture; the remaining loosely adherent clusters were used as semi-matured DCs. Cells were synchronized by serum starvation (in RPMI 1640 with 0.5% FBS) for 6 h prior to further treatment.

### Transfection and RNAi

Murine DCs were transfected with related 20~80 pmols siRNA (Santa Cruz). Briefly, transfection medium containing siRNA was directly added to the transfection reagent, gently mixed and incubated 15~45 minutes. Then, the cells were washed with transfection medium and gently overlayed with the mixture of transfection reagent and duplex siRNA. 7 h incubation after transfection, normal growth medium containing 20% FBS was appended and the cells were incubated for an additional 18~24 h. The effects of indicative siRNA in DCs were validated in Supplementary Data. The following siRNA were used: MR (sc-45361), TLR4 (sc-40261), MyD88 (sc-35987), IRAK4 (sc-45401) and control siRNA (sc-37007). However, all siRNA sequences are not provided by Santa Cruz, as stated in their datasheets: “siRNA (m) is a pool of 3 target-specific 19-25 nt siRNAs designed to knock down gene expression”.

### Lentiviral infection of primary murine DCs

Lentiviral infection of primary murine DCs was performed using MR shRNA (m) lentiviral particles (Santa Cruz: sc-45361-v) and control lentiviral particles (sc-108080). Briefly, semi-matured DCs (cultured for 4 days) were treated with Polybrene (8 μg/ml) (sc-134220). Then, the cells were infected lentiviral particles and incubated overnight. The cells were collected and used for assays 72 h later. The effect of MR shRNA (m) lentiviral particles (sc-45361-v) was validated in Supplementary Data. However, the target sequences for MR are not provided by Santa Cruz, as stated in their datasheets: “shRNA (m) Lentiviral Particles is a pool of concentrated, transduction-ready viral particles containing 3 target-specific constructs that encode 19~25 nt (plus hairpin) shRNA designed to knock down gene expression.”.

### Semi-matured DCs treatment

To determine the effects of nicotine on the expressions of MR and TLR4, endocytosis, antigenic translocation and cross-presentation, semi-matured DCs were exposed to nicotine (10^−7^ mol/l) for 12 to 16 hours. To elucidate the mechanism of nicotine-increased surface molecules expression, DCs was conferred 10 μmol/l LY294002, wortmannin, rapamycin or LY2584702 2 h prior to nicotine exposure.

### Flow cytometric measurements

The effects of nicotine on surface molecules expression, endocytosis, and cross-presentation in DCs were determined via flow cytometry [11]. Flow cytometry was done with FACSCalibur and data were analyzed with CellQuest software.

### Immunofluorescence/confocal microscope

The murine semi-matured DCs were conferred siRNA transfection prior to nicotine (10^−7^ mol/l) 12~15 hrs' stimulation. Then, the cells was conferred endotoxin-free EndoGrade-ovalbumin (50 μg/ml) 60 min pulse with or without short period (20 min) LPS (1 ng/ml) stimulation. Coverslips were fixed in 2% PFA. Cells were then permeabilized with 0.2% saponin, washed, and stained with primary antibodies over-night at 4°C. Finally, fluorescence-conjugated secondary antibodies were incubated for 1 h at 37°C. DAPI counterstaining was performed to visualize cell nuclei. Images were acquired on a Olympus FluoView FV1000 microscope with a oil immersion objective at the wavelength of 488 nm.

### Ag-specific T cell proliferation assays

Antigen-specific proliferation assays were performed as previous description [11]. Briefly, semi-matured DCs were conferred silencing prior to nicotine (10^−7^ mol/l) 12~15 hrs' stimulation. Then, the DCs were further conferred with endotoxin-free EndoGrade-ovalbumin (50 μg/ml) 6 h pulsed with or without short period (3 h) LPS (1 ng/ml) stimulation and used as stimulator cells. Responder cells were prepared by the depletion of red blood cells from splenocytes of same H-2 background C57BL/6 mice. Stimulator cells were mixed with responders at a ratio of 1:10 in 200 μl volume. After 5 d co-culture, Ag-specific T cell proliferation was determined via BrdU cell proliferation assays.

### IL-12 enzyme-linked immunosorbent assays

To investigate the effects of α7 nAChR activation on T cell differentiation, supernatants from co-cultured DC-T cells was collected and the concentration of IL-12 in the supernatants was determined by Enzyme-linked immunosorbent assay (ELISA) according to the manufacturer's guideline.

### Ag-specific IFN-γ elispot assays

To investigate the roles of MR and endosomal translocation of TAP in nicotine-increased cross-priming, antigen-specific IFN-γ Elispot assays were performed [11]. Briefly, 5×10^5^ semi-matured DCs were conferred silencing prior to nicotine (10^−7^ mol/l) exposure. Then, the DCs were pulsed with endotoxin-free EndoGrade-ovalbumin (50 μg/ml) for 6 h with or without short period (3 h) LPS (1 ng/ml) stimulation. After that, 1×10^4^ DCs were intraperitoneally transferred into C57BL/6 mice. 7 d after adoptive transfer, splenocytes of recipient were prepared and transferred into IFN–γ antibody pre-coated plate (5×10^5^ cells per well). After that, the splenocytes were further re-stimulated with peptide (SIINFEKL) at 2 μg/ml for 16~20 h. The Elispot data were presented as Spot Forming Units per million cells.

### SIINFEKL-H_2_Kb pentamers staining

Antigen-specific CD8^+^ CTL assays were performed by flow cytometry using SIINFEKL-H_2_Kb pentamers staining. Briefly, centrifuged Pro5® Pentamer at 14,000×g for 5~10 minutes. 1~2×10^6^ splenocytes was allocated, resuspended in the residual volume (~50μl), mixed with 10 μl labeled pentamers and incubated at room temperature for 10 minutes. After twice washes, the cells were performed with CD8 antibody staining. Flow cytometry was done with FACSCalibur to collect up to 500,000 events and data were analyzed with CellQuest software.

### Quantitative real time PCR

The expressiones of MR, TLR4 in DCs were investigated by RT-qPCR analysis. Briefly, total RNA was isolated from cells. Reverse transcription was performed using PrimeScript Reverse Transcriptase kit (Takara) and cDNA was used for subsequent real-time PCR reactions. Quantitative real-time PCR was conducted on an ABI Prism 7500 instrument using the Maxima SYBR green qPCR Master Mix (Takara). The cycling parameters were 95°C for 30 s, followed by 40 cycles of 95°C for 5 s, 60°C for 34 s; Each assay was performed in triplicate, and the relative expression levels (defined as fold changes) of the target genes were normalized. The following primers were used (Santa Cruz): β-actin (sc-108070-PR), MR (sc-45361-PR), TLR4 (sc-40261-PR), MyD88 (sc-35987-PR) and IRAK4 (sc-45401-PR). However, primer sequences are not provided by Santa Cruz, as stated in their datasheets: “Semi-quantitative RT-PCR may be performed to monitor gene expression knockdown using RT-PCR Primer: β-Actin (m)-PR: sc-108070-PR (600 bp); CD206 (m)-PR: sc-45361-PR (498 bp) ; TLR4 (m)-PR: sc-40261-PR (434 bp); MyD88 (m)-PR: sc-35987-PR (545 bp); IRAK-4 (m)-PR: sc-45401-PR (491 bp)”.

### Western blots

For analysis the expression of MR and TLR4, total cell lysates from nicotine-treated DCs were subjected to 7% SDS-PAGE. Proteins were transferred onto a PVDF membrane (Millipore). Membranes were blocked with 5% evaporated milk in Tris base SDS-0.05% Tween and were incubated with primary antibodies and peroxidase-conjugated secondary antibodies. Bound antibodies were revealed using the ECL western blot reagents (Advansta, CA) according to the manufacturer's directions. β-actin was used as a loading control.

### Statistical analysis

All data were expressed as average of experimental data points, and standard error means were determined using the calculated standard deviation of a data set divided by the number of data points within the data set. Statistical significance was assessed by Student's *t*-test, one-way ANOVA with the Newman-Keuls post test, with a value of p<0.05 considered statistically significant. No randomization or exclusion of data points was used. No “blinding” of investigators was done. Sample sizes were chosen according to previous experience and preliminary studies to ensure adequate power.

## SUPPLEMENTARY FIGURES



## References

[1] Mellman I, Steinman RM (2001). Dendritic cells: specialized and regulated antigen processing machines. Cell.

[2] Seya T, Shime H, Takeda Y, Tatematsu M, Takashima K, Matsumoto M (2015). Adjuvant for vaccine immunotherapy of cancer - focusing on Toll-like receptor 2 and 3 agonists for safely enhancing antitumor immunity. Cancer Sci.

[3] Villadangos JA, Heath WR, Carbone FR (2007). Outside looking in: the inner workings of the cross-presentation pathway within dendritic cells. Trends Immunol.

[4] Wessler IK, Kirkpatrick CJ (2001). The non-neuronal cholinergic system: an emerging drug target in the airways. Pulm Pharmacol Ther.

[5] Printz C (2011). Gap narrows in African American smoking-related cancers, increases in breast and colorectal cancers. Cancer.

[6] Li S, Peng Q, Chen Y, You J, Chen Z, Deng Y, Lao X, Wu H, Qin X, Zeng Z (2013). DNA repair gene XRCC1 polymorphisms, smoking, and bladder cancer risk: a meta-analysis. PLoS One.

[7] Gao FG, Wan DF, Gu JR (2007). Ex vivo nicotine stimulation augments the efficacy of therapeutic bone marrow-derived dendritic cell vaccination. Clin Cancer Res.

[8] Gao FG, Li HT, Li ZJ, Gu JR (2011). Nicotine stimulated dendritic cells could achieve anti-tumor effects in mouse lung and liver cancer. J Clin Immunol.

[9] Jin HJ, Li HT, Sui HX, Xue MQ, Wang YN, Wang JX, Gao FG (2012). Nicotine stimulated bone marrow-derived dendritic cells could augment HBV specific CTL priming by activating PI3K-Akt pathway. Immunol Lett.

[10] Jin HJ, Sui HX, Wang YN, Gao FG (2013). Nicotine up-regulated 4-1BBL expression by activating Mek-PI3K pathway augments the efficacy of bone marrow-derived dendritic cell vaccination. J Clin Immunol.

[11] Wang YY, Yang YW, You X, Deng XQ, Hu CF, Zhu C, Wang JY, Gu JJ, Wang YN, Li Q, Gao FG (2015). Ex vivo nicotine stimulation augments the efficacy of human peripheral blood mononuclear cell-derived dendritic cell vaccination via activating Akt-S6 pathway. Anal Cell Pathol (Amst).

[12] Joffre OP, Segura E, Savina A, Amigorena S (2012). Cross-presentation by dendritic cells. Nat Rev Immunol.

[13] Bertholet S, Goldszmid R, Morrot A, Debrabant A, Afrin F, Collazo-Custodio C, Houde M, Desjardins M, Sher A, Sacks D (2006). Leishmania antigens are presented to CD8+T cells by a transporter associated with antigen processing- independent pathway in vitro and in vivo. J Immunol.

[14] Burgdorf S, Schölz C, Kautz A, Tampé R, Kurts C (2008). Spatial and mechanistic separation of cross-presentation and endogenous antigen presentation. Nat Immunol.

[15] Zehner M, Chasan AI, Schuette V, Embgenbroich M, Quast T, Kolanus W, Burgdorf S (2011). Mannose receptor polyubiquitination regulates endosomal recruitment of p97 and cytosolic antigen translocation for cross-presentation. Proc Natl Acad Sci U S A.

[16] Zehner M, Burgdorf S (2013). Regulation of antigen transport into the cytosol for cross-presentation by ubiquitination of the mannose receptor. Mol Immunol.

[17] Ackerman AL, Kyritsis C, Tampé R, Cresswell P (2003). Early phagosomes in dendritic cells form a cellular compartment sufficient for cross presentation of exogenous antigens. Proc Natl Acad Sci U S A.

[18] Kopp E, Medzhitov R (2003). Recognition of microbial infection by Toll-like receptors. Curr Opin Immunol.

[19] Weck MM, Grünebach F, Werth D, Sinzger C, Bringmann A, Brossart P (2007). TLR ligands differentially affect uptake and presentation of cellular antigens. Blood.

[20] Zehner M, Marschall AL, Bos E, Schloetel JG, Kreer C, Fehrenschild D, Limmer A, Ossendorp F, Lang T, Koster AJ, Dübel S, Burgdorf S (2015). The translocon protein Sec61 mediates antigen transport from endosomes in the cytosol for cross-presentation to CD8(+) T cells. Immunity.

[21] Hu SX, Sui HX, Jin HJ, Ni XY, Liu XX, Xue MQ, Zhang Y, Gao FG (2012). Lipopolysaccharide and dose of nicotine determine the effects of nicotine on murine bone marrowderived dendritic cells. Mol Med Rep.

[22] Schwandt T, Schumak B, Gielen GH, Jüngerkes F, Schmidbauer P, Klocke K, Staratschek-Jox A, van Rooijen N, Kraal G, Ludwig-Portugall I, Franken L, Wehner S, Kalff JC (2012). Expression of type I interferon by splenic macrophages suppresses adaptive immunity during sepsis. EMBO J.

[23] Hotchkiss RS, Coopersmith CM, McDunn JE, Ferguson TA (2009). The sepsis seesaw: tilting toward immunosuppression. Nat Med.

[24] Burgdorf S, Kautz A, Böhnert V, Knolle PA, Kurts C (2007). Distinct pathways of antigen uptake and intracellular routing in CD4 and CD8 T cell activation. Science.

[25] Ackerman A, Giodini A, Cresswell P (2006). A role for the endoplasmic reticulum protein retrotranslocation machinery during cross-presentation by dendritic cells. Immunity.

[26] Nair-Gupta P, Baccarini A, Tung N, Seyffer F, Florey O, Huang Y, Banerjee M, Overholtzer M, Roche PA, Tampé R, Brown BD, Amsen D, Whiteheart SW (2014). TLR signals induce phagosomal MHC-I delivery from the endosomal recycling compartment to allow cross-presentation. Cell.

[27] Lee YR, Yang IH, Lee YH, Im SA, Song S, Li H, Han K, Kim K, Eo SK, Lee CK (2005). Cyclosporin A and tacrolimus, but not rapamycin, inhibit MHCrestricted antigen presentation pathways in dendritic cells. Blood.

[28] Bazan SB, Geginat G, Breinig T, Schmitt MJ, Breinig F (2011). Uptake of various yeast genera by antigen-presenting cells and influence of subcellular antigen localization on the activation of ovalbumin-specific CD8 T lymphocytes. Vaccine.

[29] Schuette V, Burgdorf S (2014). The ins-and-outs of endosomal antigens for cross-presentation. Curr Opin Immunol.

[30] Saveanu L, Carroll O, Weimershaus M, Guermonprez P, Firat E, Lindo V, Greer F, Davoust J, Kratzer R, Keller SR, Niedermann G, van Endert P (2009). IRAP identifies an endosomal compartment required for MHC class I cross-presentation. Science.

[31] Goldszmid RS, Coppens I, Lev A, Caspar P, Mellman I, Sher A (2009). Host ER-parasitophorous vacuole interaction provides a route of entry for antigen cross-presentation in Toxoplasma gondii-infected dendritic cells. J Exp Med.

[32] Béla SR, Dutra MS, Mui E, Montpetit A, Oliveira FS, Oliveira SC, Arantes RM, Antonelli LR, McLeod R, Gazzinelli RT (2012). Impaired innate immunity in mice deficient in interleukin-1 receptor-associated kinase 4 leads to defective type 1 T cell responses, B cell expansion, and enhanced susceptibility to infection with Toxoplasma gondii. Infect Immunol.

[33] Oliveira FS, Carvalho NB, Brandão AP, Gomes MT, de Almeida LA, Oliveira SC (2011). Interleukin-1 receptor-associated kinase 4 is essential for initial host control of Brucella abortus infection. Infect Immunol.

[34] Rauen J, Kreer C, Paillard A, van Duikeren S, Benckhuijsen WE, Camps MG, Valentijn AR, Ossendorp F, Drijfhout JW, Arens R, Burgdorf S (2014). Enhanced cross-presentation and improved CD8+ T cell responses after mannosylation of synthetic long peptides in mice. PLoS One.

[35] Fukui R, Saitoh S, Matsumoto F, Kozuka-Hata H, Oyama M, Tabeta K, Beutler B, Miyake K (2009). Unc93B1 biases Toll-like receptor responses to nucleic acid in dendritic cells toward DNA- but against RNA-sensing. J Exp Med.

[36] Kagan JC, Su T, Horng T, Chow A, Akira S, Medzhitov R (2008). TRAM couples endocytosis of Toll-like receptor 4 to the induction of interferon-beta. Nat Immunol.

[37] Tan Q, Majewska-Szczepanik M, Zhang X, Szczepanik M, Zhou Z, Wong FS, Wen L (2014). IRAK-M deficiency promotes the development of type 1 diabetes in NOD mice. Diabetes.

[38] Jeyanathan M, McCormick S, Lai R, Afkhami S, Shaler CR, Horvath CN, Damjanovic D, Zganiacz A, Barra N, Ashkar A, Jordana M, Aoki N, Xing Z (2014). Pulmonary M. tuberculosis infection delays Th1 immunity via immunoadaptor DAP12-regulated IRAK-M and IL-10 expression in antigen-presenting cells. Mucosal Immunol.

[39] Blander JM, Medzhitov R (2004). Regulation of phagosome maturation by signals from toll-like receptors. Science.

[40] Dinter J, Gourdain P, Lai NY, Duong E, Bracho-Sanchez E, Rucevic M, Liebesny PH, Xu Y, Shimada M, Ghebremichael M, Kavanagh DG, Le Gall S (2014). Different antigen-processing activities in dendritic cells, macrophages, and monocytes lead to uneven production of HIV epitopes and affect CTL recognition. J Immunol.

[41] Ezekowitz RA, Sastry K, Bailly P, Warner A (1990). Molecular characterization of the human macrophage mannose receptor: demonstration of multiple carbohydrate recognition-like domains and phagocytosis of yeasts in Cos-1 cells. J Exp Med.

[42] Keler T, Ramakrishna V, Fanger MW (2004). Mannose receptor-targeted vaccines. Expert Opin Biol Ther.

