# Supplementary material for: Increased translocation of antigens to endosomes and TLR4 mediated endosomal recruitment of TAP contribute to nicotine augmented cross-presentation
Source: Oncotarget. 2016 May 20;7(25):38451–66. doi: 10.18632/oncotarget.9498 (PMC5122403; doi:10.18632/oncotarget.9498)
Supplement: Supplementary file 1 [file oncotarget-07-38451-s001.pdf]

SUPPLEMENTARY FIGURES

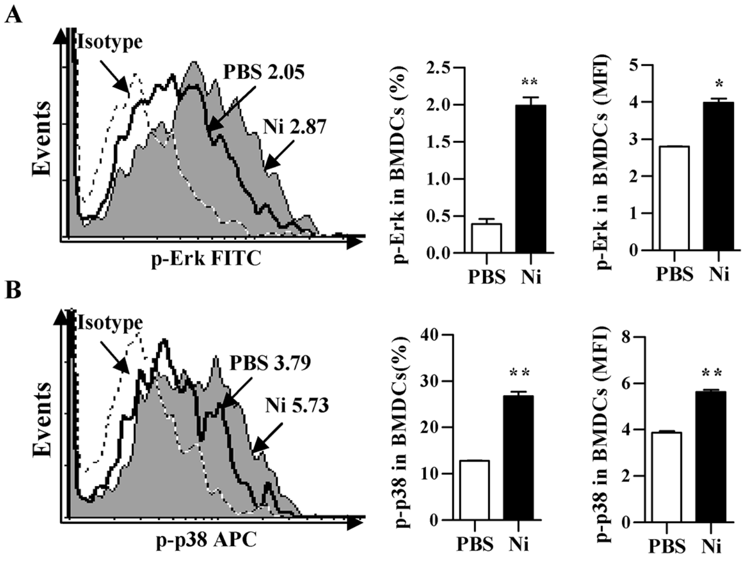

Supplementary Figure S1: The treatment with nicotine induces Erk-p38 activation in murine semi-mature DCs. A-B. Murine bone marrow-derived semi-mature DCs were stimulated with nicotine (10^{-7} mol/l) and the phosphorylation of Erk (A) and p38 (B) was determined by flow cytometry. One representative from 3 independent experiments is shown. Numbers in histogram indicates mean fluorescence intensity (MFI) of test samples. Data were given as mean ± SEM, Student t test, * p<0.05, ** p<0.01. Ni: nicotine.

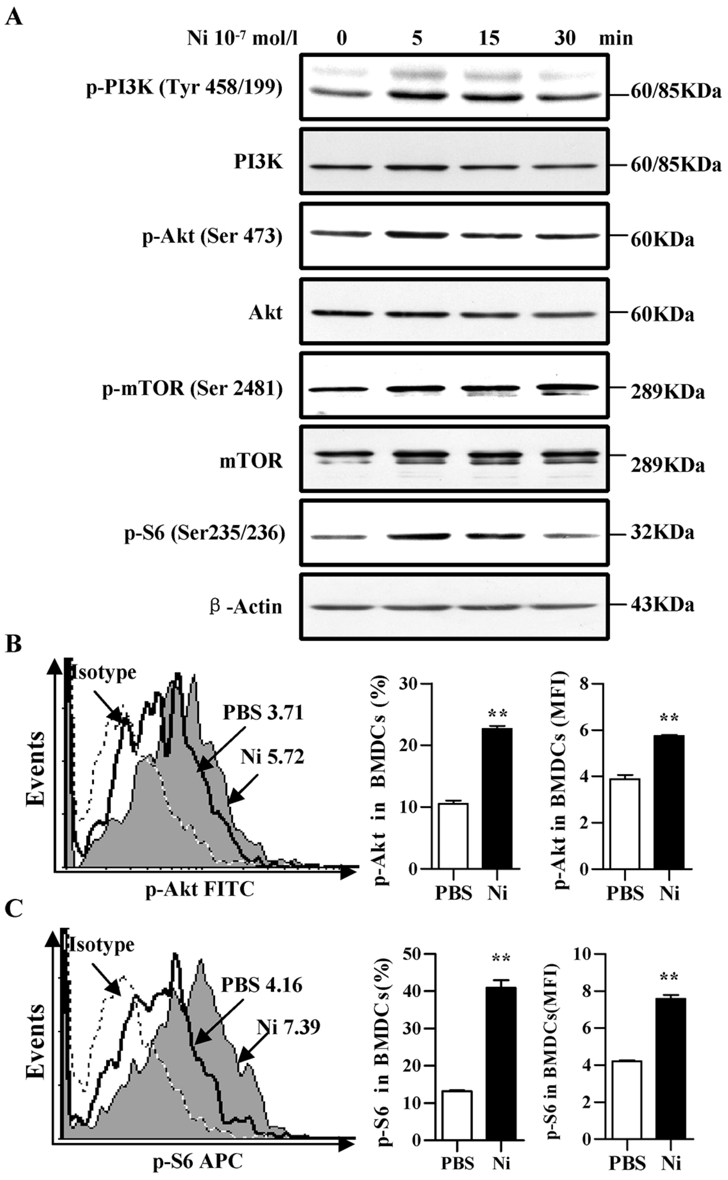

Supplementary Figure S2: The treatment with nicotine induces PI3K-Akt-mTOR-p70S6 activation in murine semi-mature DCs. A-C. Murine bone marrow-derived semi-mature DCs were stimulated with nicotine (10^{-7} mol/l) and the phosphorylation of PI3K, Akt, mTOR and p70S6 was determined via western blot analyses (A) and flow cytometry (B-C). One representative from 3 independent experiments is shown. β-actin was used as an internal control (A). Numbers in histogram indicates mean fluorescence intensity (MFI) of test samples (B-C). Data were given as mean ± SEM, Student t test, ** p<0.01. Ni: nicotine.

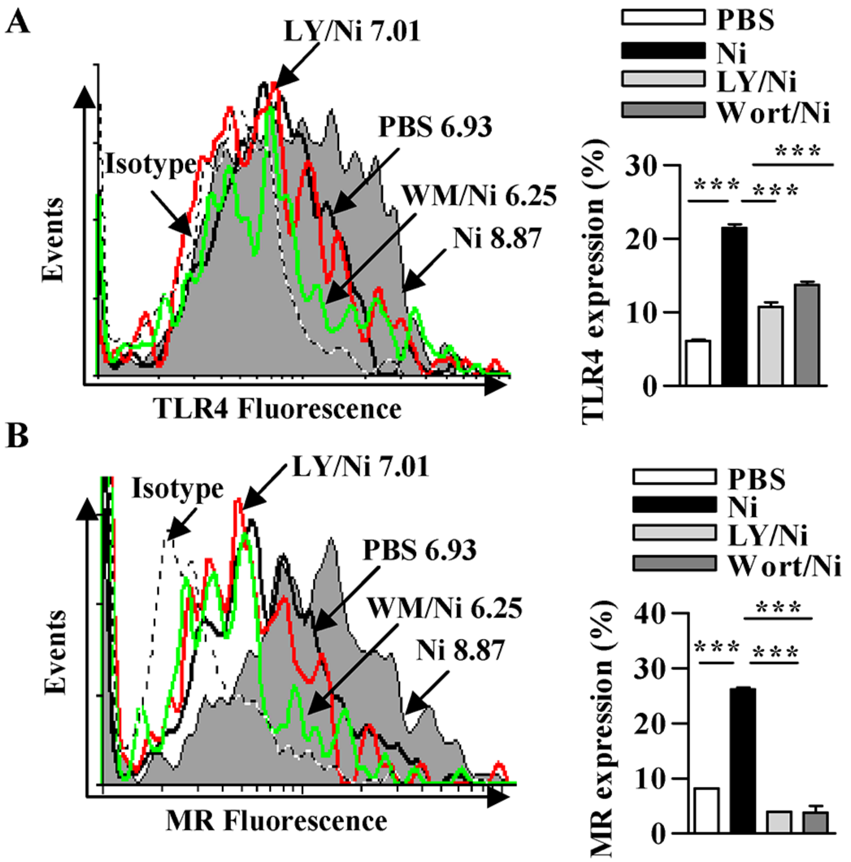

Supplementary Figure S3: Nicotine up-regulates mannose receptor and TLR4 expression via PI3K-Akt pathway in human PBMC-derived DCs. A-B. DCs derived from human PBMC with recombinant human GM-CSF and IL-4 were pretreated with LY294002 (10 µmol/l) or wortmannin (10 µmol/l) 2 h prior to nicotine (10^{-7} mol/l) 12~15 h stimulation. The expression of mannose receptor and TLR4 was determined by flow cytometry. Numbers in histogram indicates MFI of analyzed population (left). Statistical analysis of positive percentages (right) is shown. The data are presented as the mean±SEM, n=3, ***p<0.001, one-way ANOVA with Newman-Keulspost test. One representative from 3 independent experiments is shown. MR: mannose receptor; Ni: nicotine; LY: LY294002; Wort: wortmannin.

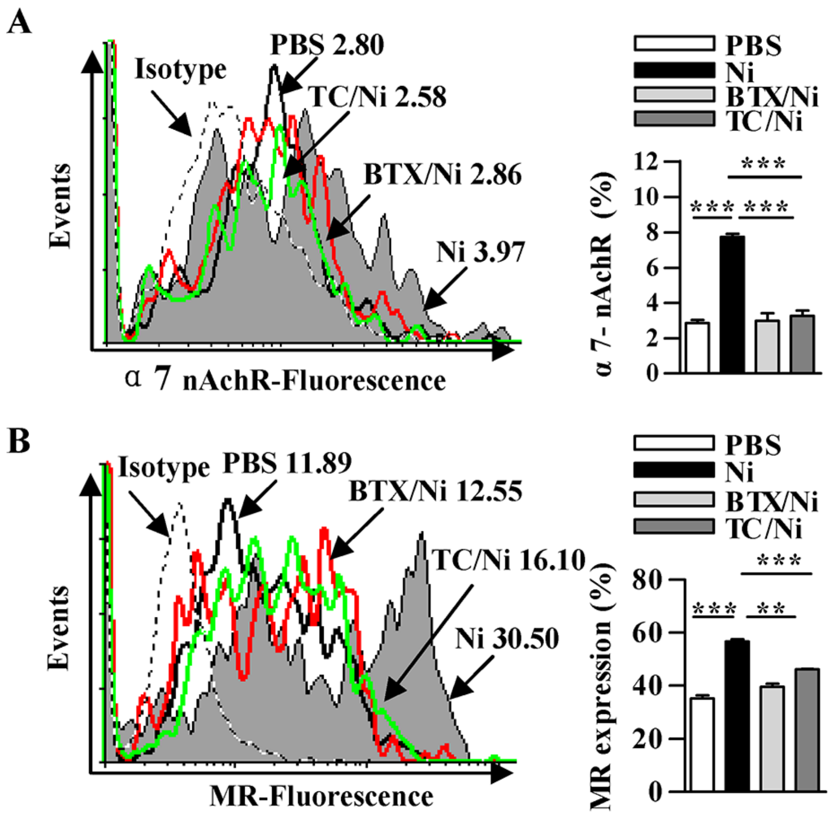

Supplementary Figure S4: Nicotine up-regulates mannose receptor expression via α7 nicotinic acetylcholine receptor in human PBMC-derived DCs. DCs derived from human PBMC with recombinant human GM-CSF and IL-4 were conferred with α-bungarotoxin (1 μg/ml) or tubocurarine chloride (4×10^{-5} mol/l) 2 h prior to nicotine (10^{-7} mol/l) 12~15 h stimulation. The expression of α7 nAChR **A.** and MR **B.** was determined by flow cytometry. Numbers in histogram indicate MFI of analyzed population (left). Statistical analysis of positive percentages (right) is shown. The data are presented as the mean±SEM, ***$p<0.001$, one-way ANOVA with Newman-Keulspost test. One representative from 3 independent experiments is shown. MR: mannose receptor; Ni: nicotine; BTX: α-bungarotoxin; TC: tubocurarine chloride; α7 nAChR: α7 nicotinic acetylcholine receptor.

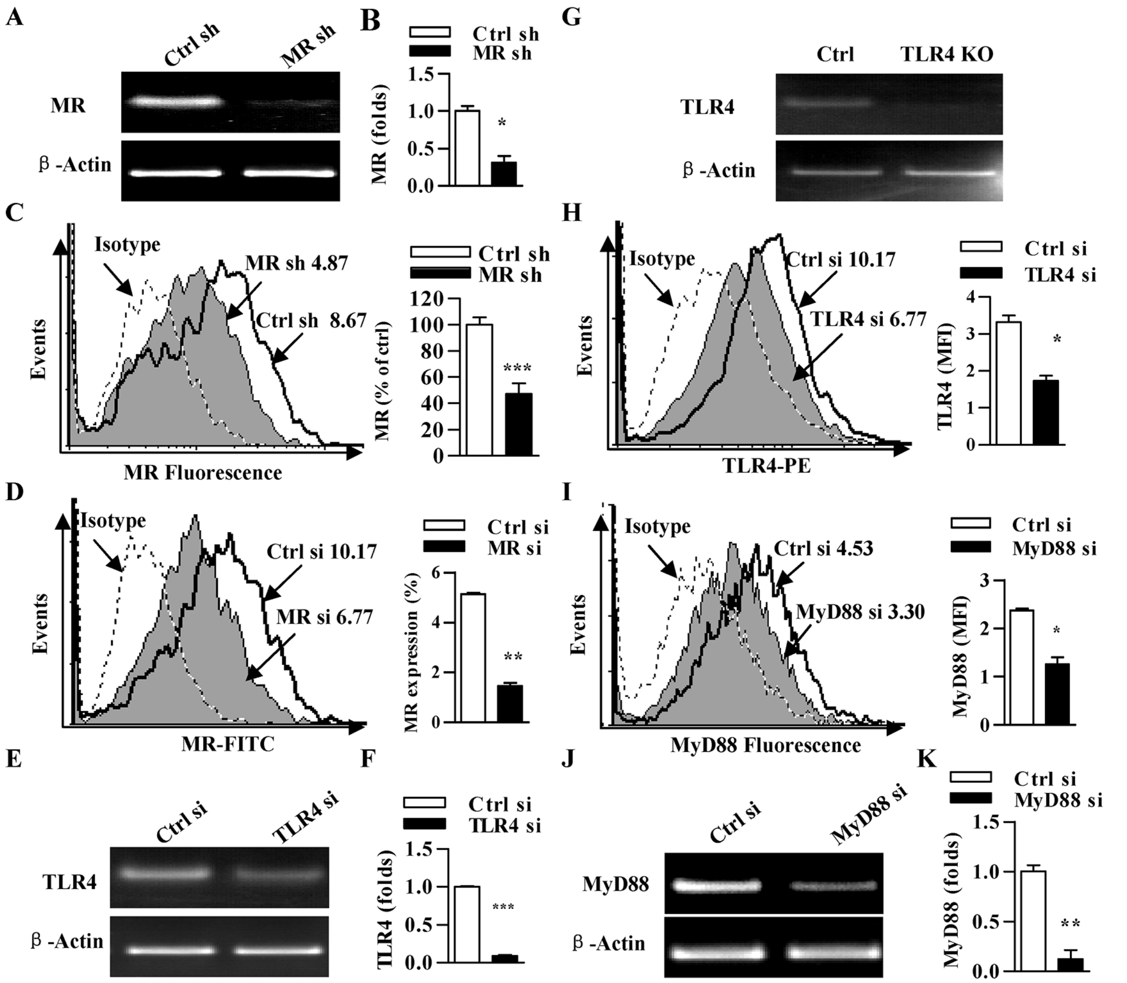

Supplementary Figure S5: The efficiency of related RNAi interference in murine DCs. Whole cellular RNA was extracted from shRNA **A-C.** / siRNA **D-F, H-K.** transfected DCs or TLR4$^{-/-}$ mouse bone marrow derived DCs (G) and reverse-transcripted to cDNA. The expression of mannose receptor (A-D), TLR4 (E-H) and MyD88 (I-K) was determined via RT-PCR (A,E,G,J), real-timePCR (B,F,K) and flow cytometric analyses (C,D,H,I). Representative results from 1 of 3 independent experiments are shown. β-actin was used as an internal control (A,E,G,J). The data are presented as the mean±SEM (B-D). * p<0.05, ** p<0.01, ***p<0.001, student t test. sh: shRNA; si: siRNA; MR: mannose receptor.

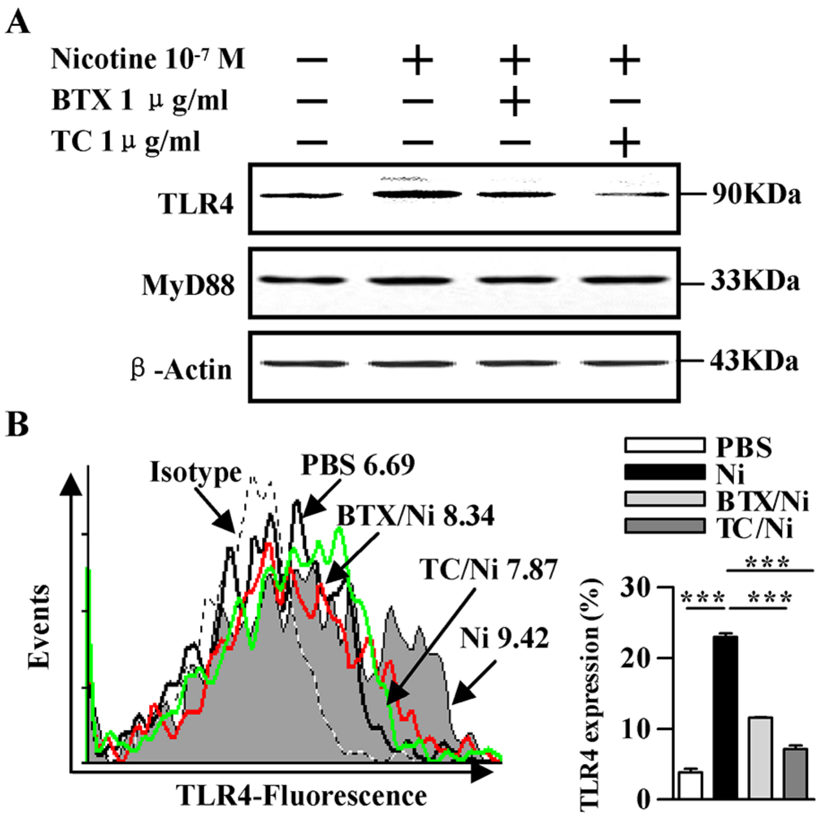

Supplementary Figure S6: Nicotine up-regulates TLR4 expression via α7 nicotinic acetylcholine receptor in murine and human DCs. DCs derived from murine bone marrow **A.** or human PBMC **B.** were conferred with α-bungarotoxin (1 μg/ml) or tubocurarine chloride (1 μg/ml) 2 h prior to nicotine (10^{-7} mol/l) 12~15 h stimulation. The effect of nicotine on the expression of MyD88 and TLR4 was determined via western blot (A) and flow cytometric analyses (B). β-actin was used as an internal control. Representative results from 1 of 3 independent experiments are shown. Ni: nicotine; BTX: α-bungarotoxin; TC: tubocurarine chloride.

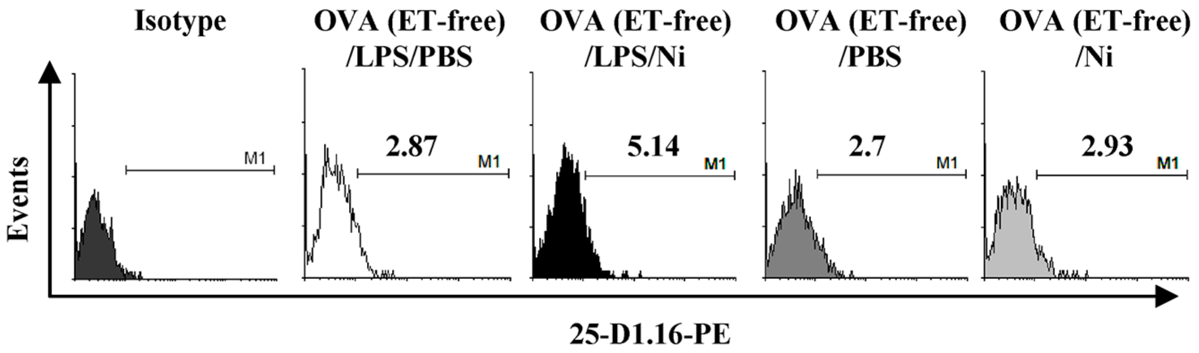

Supplementary Figure S7: Flow cytometric analyses of DCs previously exposed to 'ordinary' OVA or endotoxin-free OVA (OVA(ET-free)) with or without LPS exposure. DCs derived from murine bone marrow were conferred with nicotine (10^{-7} mol/l) 12~15 h stimulation and further incubated with endotoxin-free OVA with or without LPS exposure. Numbers in histogram plot indicate positive percentages of analyzed population. Data are representative of three independent experiments.
